# High risk for occupational exposure to HIV and utilization of post-exposure prophylaxis in a teaching hospital in Pune, India

**DOI:** 10.1186/1471-2334-8-142

**Published:** 2008-10-21

**Authors:** Amita Gupta, Shuchi Anand, Jayagowri Sastry, Anandini Krisagar, Anita Basavaraj, Shreepad M Bhat, Nikhil Gupte, Robert C Bollinger, Arjun L Kakrani

**Affiliations:** 1 Johns Hopkins University School of Medicine, Baltimore, MD, USA; 2 Brigham and Women's Hospital, Boston, MA, USA; 3 Byramji Jeejeebhoy Medical College (BJMC)-Johns Hopkins University Maternal Infant Transmission Study, Pune, India; 4 Sassoon Hospital, Byramji Jeejeebhoy Medical College (BJMC), Pune, India

## Abstract

**Background:**

The risk for occupational exposure to HIV has been well characterized in the developed world, but limited information is available about this transmission risk in resource-constrained settings facing the largest burden of HIV infection. In addition, the feasibility and utilization of post-exposure prophylaxis (PEP) programs in these settings are unclear. Therefore, we examined the rate and characteristics of occupational exposure to HIV and the utilization of PEP among health care workers (HCW) in a large, urban government teaching hospital in Pune, India.

**Methods:**

Demographic and clinical data on occupational exposures and their management were prospectively collected from January 2003–December 2005. US Centers for Diseases Control guidelines were utilized to define risk exposures, for which PEP was recommended. Incidence rates of reported exposures and trends in PEP utilization were examined using logistic regression.

**Results:**

Of 1955 HCW, 557 exposures were reported by 484 HCW with an incidence of 9.5 exposures per 100 person-years (PY). Housestaff, particularly interns, reported the greatest number of exposures with an annual incidence of 47.0 per 100 PY. Personal protective equipment (PPE) was used in only 55.1% of these exposures. The incidence of high-risk exposures was 6.8/100 PY (n = 339); 49.1% occurred during a procedure or disposing of equipment and 265 (80.0%) received a stat dose of PEP. After excluding cases in which the source tested HIV negative, 48.4% of high-risk cases began an extended PEP regimen, of whom only 49.5% completed it. There were no HIV or Hepatitis B seroconversions identified. Extended PEP was continued unnecessarily in 7 (35%) of 20 cases who were confirmed to be HIV-negative. Over time, there was a significant reduction in proportion of percutaneous exposures and high-risk exposures (p < 0.01) and an increase in PEP utilization for high risk exposures (44% in 2003 to 100% in 2005, p = 0.002).

**Conclusion:**

Housestaff are a vulnerable population at high risk for bloodborne exposures in teaching hospital settings in India. With implementation of a hospital-wide PEP program, there was an encouraging decrease of high-risk exposures over time and appropriate use of PEP. However, overall use of PPE was low, suggesting further measures are needed to prevent occupational exposures in India.

## Background

Occupational exposure to blood or other body fluids in healthcare settings constitutes a small but significant risk of transmission of HIV and other blood-borne pathogens [1,2]. In addition, such exposures can cause tremendous anxiety, fear and stress among healthcare workers (HCW) that can have a negative impact not only on the HCW, but also their families and colleagues [3]. The World Health Organization estimates that 3 million percutaneous exposures occur annually among 35 million HCW globally, with over 90% occurring in resource-contrained countries [4]. As a consequence of these exposures, an estimated 66,000 hepatitis B, 16,000 hepatitis C, and up to 1000 HIV infections occur each year.

These infections acquired through the occupational route are largely preventable through strict infection control, universal precautions, use of safe devices, proper waste disposal, immunization against hepatitis B virus, and prompt management of exposures including the use of post-exposure prophylaxis (PEP) for HIV (estimated to reduce HIV seroconversion by 81%) [5]. The use of these strategies are now the standard of care in most high-income nations and have reduced the risk of HIV and hepatitis transmission among HCW. In resource-constrained settings where the largest burden of HIV and hepatitis exist, however, there is limited surveillance and data regarding health care-related occupational exposures and the use of PEP. Furthermore, a lack of personal protective equipment (PPE), availability of safe devices, proper disposal of sharps and waste, and a high demand for injections place HCW in these settings at high risk for occupational exposures and infection [2].

India has a population of approximately 1 billion and an estimated HIV adult seroprevalence of 0.3% (2.5 million persons), a Hepatitis B surface antigen (HBsAg) positivity of 1–8% and a <1% prevalence of Hepatitis C in the general population currently [6-11]. Data specific to hospital-based prevalence of HIV, Hepatitis B and Hepatitis C are limited and vary by region in India. Prevalence for HIV is higher in hospitalized patients, than that in the general population. Limited data suggest that HCW in India may have a high frequency of occupational exposures to blood [12], are not adequately implementing universal precautions [13], are not aware of the true risk of occupational HIV transmission, and have little knowledge of PEP [14] compared to HCW in many Western settings.

To expand the understanding of this issue in resource-constrained settings, like India, we evaluated the epidemiology of occupational exposures and the utilization of a newly established PEP program among HCW in a large, urban government teaching hospital in Pune, where HIV antenatal prevalence was approximately 3.5%.

## Methods

### Setting

The Byramji Jeejeebhoy Medical College (BJMC) and Sassoon Hospital have an ongoing NIH-funded clinical trial collaboration with Johns Hopkins University School of Medicine to prevent maternal-to-infant HIV transmission. As part of this collaboration, a hospital-wide PEP program based on U.S. Centers for Disease Control and Prevention (CDC) guidelines for the management of occupational exposures to blood-borne pathogens was initiated in August 2002 [1]. This study was approved by the Johns Hopkins and the BJMC IRB and Ethics Committee, respectively.

Approximately 1,955 HCW work at Sassoon Hospital, BJMC. Within six months of the program's initiation, occupational safety training was begun for all incoming interns through the Department of Medicine and for all nurses through the ongoing NIH-sponsored trial.

At all sites gloves and masks were generally available in wards and clinics, while procedure rooms also stocked gowns. PPE, in part, was supplied by the clinical trial to ensure these were available to HCW involved with the trial when government supplies were unavailable. Posters explaining the PEP protocol with contact information in event of exposure were displayed throughout the hospital.

### Exposure management

An exposed HCW reported to the trial's medical research officer or to a designated Department of Medicine physician. This physician administered a detailed questionnaire regarding the timing and setting of exposure, assessed the severity of exposure and investigated source patient status. Using the then available 2001 CDC occupational exposures management guidelines, an exposure was defined as high-risk and warranted a stat dose of PEP if the exposure was percutaneous or mucocutaneous and 1) the source was known to be HIV-infected, 2) the source was felt to be at high risk for HIV infection, or 3) the source was unknown (e.g., needlestick exposure while removing a trash bag) or the source's risk factors for HIV infection were unknown [1].

The last criterion for high-risk exposure was formulated using CDC's recommendations to administer PEP for source of unknown HIV status or an unknown source, regardless of the severity or type of exposure, "in settings where exposure to HIV-infected persons is likely." The HIV prevalence among antenatal attendees at the study site is approximately 3.5% and is assumed to be higher in the hospitalized population. Given these prevalence rates, our clinicians felt that unless a source was specifically judged to be low risk for HIV, all cases with an unknown source or unknown source risk factors for HIV would generally merit a stat dose PEP. However, case-by-case decisions were made by clinicians who interviewed HCW.

Initial stat PEP dose was: (1) zidovudine and lamuvidine, or (2) zidovudine, lamuvidine and indinavir (both provided for free by NIH-sponsored clinical trial). Source consent for rapid HIV testing was performed during office hours at the hospital's voluntary counseling and testing center laboratory at a cost of 10 Indian rupees (INR) ($0.25 USD). During off-hours, the Department of Microbiology resident on emergency call or a trial laboratory technician performed the test. HBsAg testing was performed at a cost, with both HCW and source patients each incurring costs of INR 80–120 ($2–3 USD) per test. Each exposed HCW was offered free baseline as well as follow-up laboratory studies at the hospital's laboratory (complete blood count, liver transaminases, serum creatinine and HIV test). Hepatitis B testing was advised for non-immunized individuals and was paid for by the HCW. Standard Hepatitis B management was provided if case deemed to be at high risk exposure (immune globulin if not vaccinated or HCW status unknown and Hepatitis B vaccination if not previously vaccinated). Hepatitis C status was not assessed in source or HCW. The clinical trial pharmacist or laboratory technician dispensed stat HIV PEP doses. Prescriptions for extended PEP (total 28-day course) were refilled every seven days through the clinical trial pharmacy if the treating clinician recommended it. Extended PEP regimens were either zidovudine and lamuvidine, or zidovudine, lamuvidine and indinavir.

### Data Collection

Beginning in January 2003, demographic and clinical data were collected using a standardized questionnaire on all reported exposures. Data collected included age, sex, occupation, department and Hepatitis B vaccination status of HCW. HCW were asked about the type of exposure (needle, laceration or splash), use of PPE and activity during exposure. The treating physician then categorized the exposure as more or less severe according to CDC guidelines [1].

The exposed HCW also facilitated the assessment of source, reporting on source location and HIV status if known. Whenever possible, the treating physician attempted to directly evaluate the source's risk factors for HIV and categorized them as high or low risk for HIV infection. When a direct assessment was not possible, the physician relied on the reporting HCW if he/she was a physician or used the severity of exposure as the only criterion for subsequent interventions. For HCW who were prescribed extended PEP regimens, pharmacy records of their prescriptions were used to track the type of regimen prescribed and the number of days it was used.

The initial January 2003 questionnaire was modified in April 2004 to collect the following additional data: exact time interval from exposure to contact with designated physician.

### Statistical Analysis

We analyzed data collected from January 2003 to December 2005. All statistical analyses were performed with the use of STATA version 9.1 (College Station, TX) and SPSS (Chicago, version 14.0). Person-time was calculated assuming that each HCW worked all year round as vacation time is minimal (<2 weeks per year). In addition, there were effectively the same number of faculty, housestaff and nursing staff per year throughout the study period and approximately the same number of ancillary hospital staff so we assumed a constant number of 1955 HCW per year. Correlates of high-risk exposure were examined using chi-square tests for categorical variables and Mann-Whitney tests for continuous variables. More detailed information was available on extended PEP from January 2005 to February 2006 permitting additional analyses of PEP compliance during this time period. We also performed logistic regression using time as a linear variable was performed to assess trends in proportions of exposures and behavior types over the time period between 2002 and 2005. Lastly, multivariable logistic regression was used to identify factors that were independently associated with inappropriate prescription of PEP in cases of low HIV risk exposure. We used the PEP stat dose exposure variable as the outcome and assessed age, sex, occupation, department, exposure type, use of personal protective equipment, visible blood, wound cleaned stat, source status, year of exposure, and exposure activity. Factors that were significant in univariable analysis at the p < 0.20 level were considered in the final model.

## Results

### Characteristics of HCW reporting exposures

Among 1,955 HCW employed between January 2003 and December 2005, 557 occupational exposures were reported by 484 HCW. The median age of exposed HCW was 23 years and 53.2% were male (Table 1). Nearly half of the reported exposures came from the Medicine and Obstetrics/Gynecology departments. The greatest number of exposures was reported among interns (53.1%) (i.e. persons in their first year post-medical school), followed by residents (22.8%), who were in years 2 through 5 post-medical school. Sixty-two HCW reported repeat exposures during the study period; most were either interns (69.3%) or residents (19.3%). Overall average annual incidence of reported exposures among HCW was 9.5 exposures per 100 person-years (PY). Interns had the highest annual incidence at 47.0/100 PY (Table 2).

**Table 1:** Characteristics of 557 HCW reporting occupational exposures at an urban teaching hospital in Pune, India, 2003–2005

**Characteristic**	Total N = 557 (%)	High-risk^#^ N = 339 (%)	Low-risk N = 211 (%)	p
**Median Age, years (range)**	23 (18–58)	23 (18–58)	23 (18–57)	0.2
**Male**	295 (53.2)	178 (52.8)	112 (53.1)	0.33
**Occupation**				
Intern	296 (53.1)	159 (46.9)	134 (63.5)	0.0001
Resident	127 (22.8)	84 (24.8)	42 (19.9)	0.19
Nurse	42 (7.5)	28 (8.3)	13 (6.2)	0.36
Student Nurse	33 (5.9)	24 (7.1)	9 (4.3)	0.18
Other	59 (10.6)	38 (11.2)	11 (5.2)	0.02
**Department**				
Medicine	150 (26.9)	101 (29.8)	49 (23.2)	0.09
Obstetrics/Gynecology	132 (23.7)	67 (19.8)	63 (29.9)	0.008
Casualty (emergency room)	96 (17.2)	49 (14.5)	46 (21.8)	0.03
Surgery	84 (15.1)	57 (16.8)	26 (12.3)	0.15
Pediatrics	30 (5.4)	16 (4.7)	14 (6.6)	0.34
Other	58(10.4)	44 (13.0)	11 (5.2)	0.003
**Median time between exposure and reporting (n = 329) hours:minutes(range)**	0:30 (0–122:30)	0:30 (0–122:30)	0:30 (0–23:56)	0.88
Reporting within 24 hours	313 (95.1)	149 (97.3)	164 (100)	0.56
**Wound cleaned stat**	523 (93.9)	314 (92.6)	203 (96.2)	0.13
**Personal protective equipment used**	307 (55.1)	182 (53.7)	124 (58.8)	0.25
Gloves	279 (50.1)	165 (48.7)	113 (53.6)	0.26
Mask	52 (9.3)	31 (9.1)	20 (9.5)	0.89
Gown	51 (9.2)	30 (8.8)	20 (9.5)	0.80
Eyewear	18 (3.2)	8 (2.4)	10 (4.7)	0.13
Other	5 (0.9)	5 (1.5)	0 (0.0)	0.54
**Hepatitis B Vaccination/job category (% vaccinated)**	424 (76.1)	249 (73.5)	172 (81.5)	0.03
Intern	238 (80.4)	125 (78.6)	111 (82.8)	0.0003
Resident	111 (89.5)	72 (85.7)	39 (92.8)	0.43
Student Nurse	22 (66.7)	14 (58.3)	8 (88.9)	0.84
Nurse	26 (61.9)	18 (64.3)	8 (57.1)	0.41
Other	25 (42.4)	19 (50.0)	6 (54.5)	0.13
**Exposure characteristic**				
**Percutaneous**	452 (81.1)	280 (82.6)	160 (75.8)	0.05
More severe	159 (28.5)	107 (31.6)	49 (23.2)	0.03
Needle stick	420 (75.4)	256 (75.5)	152 (74.1)	0.36
Hollow needle	280 (66.7)	175 (68.4)	105 (69.1)	0.67
Solid needle	111 (26.4)	66 (25.8)	45 (29.6)	0.60
Unknown/not reported	29 (6.9)	15 (5.9)	2 (1.3)	0.02
Laceration	34 (6.1)	24 (7.3)	8 (3.9)	0.11
**Mucocutaneous**	105 (18.8)	51 (15.4)	45 (22.0)	0.06
Large volume	13 (12.4)	6 (11.8)	4 (9.8)	0.91
Small volume	91 (86.7)	45 (88.2)	41 (91.1)	0.05
**Exposed to blood**	489 (87.8)	286 (84.4)	200 (94.8)	0.0002
**Exposed worker activity**				
Handling sharp during procedure	331 (59.4)	217 (64.0)	114 (54.0)	0.02
Handling sharp after procedure	98 (17.6)	58 (17.1)	40 (19.0)	0.58
Recapping	46 (8.3)	30 (8.8)	16 (7.6)	0.60
Disposing equipment	30 (5.4)	12 (3.5)	18 (8.5)	0.01
Sharp left around/not safely disposed	31 (5.6)	5 (1.5)	8 (3.8)	0.08
Other	21 (3.8)	8 (2.4)	13 (6.2)	0.02

*Not all percentages add to 100% as some data were missing

^#^High risk exposure defined as an exposure for which PEP is recommended and includes: (1) cases in which source tested or was known to be HIV positive, (2) cases where source was judged to be at high risk for HIV by clinicians, and (3) cases where source status and/or risk for HIV remained unknown.

**Table 2:** Average incidence of exposures by job category per 100 person-years (PY) at an urban teaching hospital in Pune, India 2003–2005.

**Job category**	Number employed*	**Total exposures per 100 PY**	**Percutaneous exposures per 100 PY**	**Mucocutaneous exposures per 100 PY**	High-risk exposure per 100 PY**
Interns	210	47.0	38.9	8.1	25.2
Residents	300	14.1	10.8	3.3	9.3
Student Nurses	120	9.4	6.1	3.1	6.7
Nurses	703	2.0	0.3	1.7	1.3
Other	622	3.1	2.8	0.4	2.0
Total	1955	9.5	7.7	1.8	6.8

*Number of employees per year in each job category was assumed to have remained stable over the 3-year period.

**High risk exposure defined as an exposure fro which PEP is recommended according to US CDC guidelines (1) and includes: (1) cases in which source tested or was known to be HIV positive, (2) cases where source was judged to be at high risk for HIV by clinicians, and (3) cases where source status and/or risk for HIV remained unknown.

Ninety-five percent of HCW reported their exposure within 24 hours; the median time between exposure and reporting was 30 minutes. Fifty-five percent of HCW reported using PPE at the time of their exposure. Gloves were used in 50.1% of all exposures, while 9.3% reported use of a mask. Baseline Hepatitis B vaccination rate was 79.1%. Housestaff were more likely to have been vaccinated than others (82.5% vs. 54.5%, p < 0.0001).

### Exposure Description

Of 557 exposures, 452 (81.1%) exposures were percutaneous (75.1% were needlesticks and 6.1% were lacerations) and 105 (18.8%) were mucocutaneous (Table 1). A total of 429 (77.0%) of exposures were due to handling a sharp during or after a procedure; recapping specifically accounted for 46 (8.3%) of exposures. Percutaneous exposures were the most common across all job categories except for nurses for whom mucocutaneous exposures were more common (Table 2).

### High-risk exposures

The incidence of high-risk exposures (PEP recommended according to CDC guidelines) was 6.8/100 PY. A majority (83.8%) of exposures were percutaneous and 49.2% incidents occurred during a procedure or disposing of equipment. Interns and residents made up 46.9% and 24.8% of these exposures, respectively.

### Source Description

The overall HIV prevalence of known source patients was 15.8%. Source status was known prior to exposure in 101 (18.1%) cases, 73 (72.3%) of whom were HIV-infected. For 456 cases where source status was unknown prior to testing, the clinicians judged 75 (13.4%) sources to be high risk for HIV. Seven (9.3%) of these 75 tested HIV-positive compared to 5 (2.6%) of 188 cases judged to be at low risk for HIV and 3 (15%) of 20 determined to be unknown risk (p = 0.02).

As a result of HIV testing, 15 additional sources were identified as HIV-positive, with rapid HIV antibody testing capturing 13 cases. Although ELISA was recommended for all sources whose status was unknown prior to testing, only 26 (5.7%) had them performed. In contrast, 320 (57.5%) source patients had rapid HIV antibody tests completed. The source status remained unknown for 173 (31.1%) of exposures.

### Exposure Management and PEP utilization

**Figure 1:**
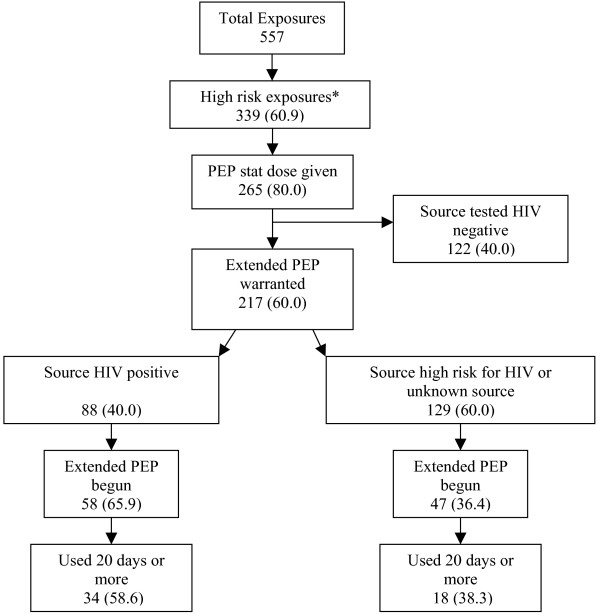
**PEP extended regimen use: Clinical decisions and HCW compliance**. *High risk exposure defined as an exposure for which PEP is recommended according to US CDC guidelines (1) and includes: 1) cases in which source tested or was known to be HIV positive, 2) cases where source was judged to be at high risk for HIV by clinicians, and 3) cases where source status and/or risk for HIV remained unknown.

A stat PEP dose was given for 401 (72.0%) exposures; 265 (80.0%) of 339 high-risk exposures and 136 (62.4%) of 218 other exposures (Figure 1). For 72 (81.8%) of 88 exposures where the source was determined to be HIV-infected, the HCW received a stat dose.

Excluding sources that ultimately tested HIV-negative in the high-risk exposures, 217 (64.0%) cases warranted extended PEP regimen based on CDC guidelines. When the source status was confirmed to be HIV-positive, 58 (65.9%) HCW began and 34 (58.6%) completed more than 20 days of a PEP regimen. In 129 high-risk cases where source status remained unknown, 47 (36.4%) initiated extended PEP, 18 (38.3%) completed more than 20 days, and 11 (23.4%) stopped due to intolerance to PEP regimen. PEP extended regimen was begun on 20 (16.1%) cases where the source was confirmed to be HIV-negative, and in 7 (35.0%) the regimen was taken for 20 or more days.

Tetanus vaccination was administered to 113 persons who could not recall tetanus vaccination or had not had it in the past 5 years and Hepatitis B vaccine was given to 67 of 112 persons who had not previously received it. Ten HCW received Hepatitis B immune globulin because they were considered to have a high risk exposure to Hepatitis B. There were no documented or reported HIV or Hepatitis B seroconversions during the study period.

### Factors associated with PEP initiation in context of low risk HIV exposures

Of 557 exposures, 218 were defined as low risk HIV exposures based on CDC guidelines and 136 (24.4%) of these received a stat PEP dose. The factors independently associated with receiving stat PEP despite having a low risk HIV exposure were type of exposure other than needlestick (e.g. splash) [adjusted odds ratio (AOR) 2.9, 95% CI 1.1–7.9], source HIV status unknown (AOR 6.1, 95% CI 2.5–15.0), exposure occurred when sharp was left around or in trash (AOR 4.4, 95% CI 1.9–9.9), or blood was visible (AOR 1.9, 95% CI 1.1–3.6).

### Trends over time

**Figure 2:**
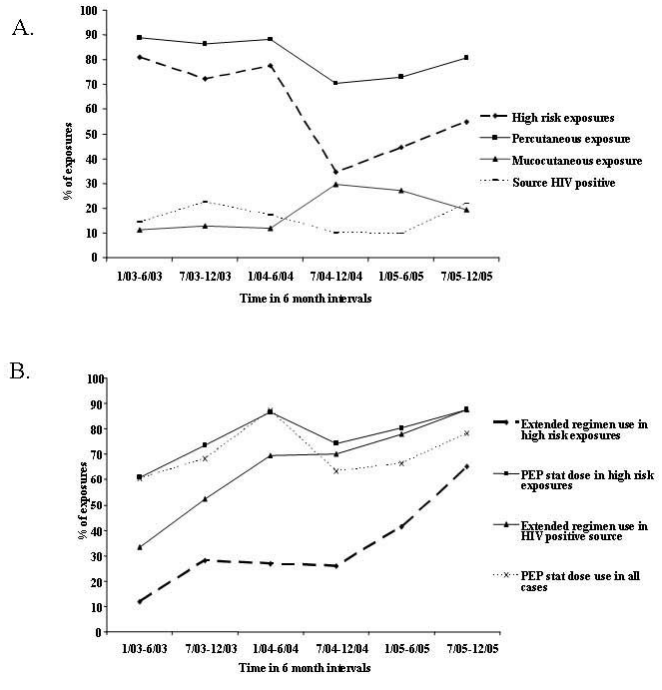
**A. Trends over time in exposure types in a large teaching hospital in Pune, India, 2003–2005.** B. Trends over time in PEP stat dose and extended regimen utilization types in a large teaching hospital in Pune, India, 2003–2005. *High risk exposure defined as an exposure fro which PEP is recommended according to US CDC guidelines (1) and includes: (1) cases in which source tested or was known to be HIV positive, (2) cases where source was judged to be at high risk for HIV by clinicians, and (3) cases where source status and/or risk for HIV remained unknown.

Over a 3-year period, percutaneous exposures decreased significantly over time (p = 0.002) as did high-risk exposures (p < 0.001) (Figure 2A). Mucocutaneous exposures, however, increased over time (p = 0.002). The proportion of sources known HIV-positive prior to testing did not change significantly over time, nor did use of PPE or hepatitis B vaccination change over time (p > 0.05).

Overall the utilization of PEP stat doses did not increase over time but recommended use of PEP stat doses for high risk exposures did increase significantly over time (44% in 2003 to 100% in 2005, p = 0.002) (Figure 2B). The odds of receiving extended PEP regimen for high-risk exposures increased significantly over time (OR 1.24; 95% CI 1.09–1.42; p = 0.001).

## Discussion

Our prospective study at a large public teaching hospital in India highlights the high incidence of occupational exposures, particularly among medical housestaff. We found that only half of the exposures involved use of PPE. While recapping contributed to exposures, handling sharps such as IV needles and sutures during a procedure or after a procedure were the most common reason for an exposure. Hepatitis B vaccination, which was highest among medical housestaff and lowest among ancillary hospital staff, nevertheless was higher than that reported in many other resource-constrained settings [15-17]. In the setting of HCW education and a structured PEP program, the reported number of percutaneous and high risk exposures decreased over time and the utilization and appropriate implementation of PEP increased over time.

Only a few studies have been published regarding occupational exposures in India. A study at a private non-teaching hospital in Mumbai found that over a six-year period, 380 needlestick injuries were reported [18]. Nurses reported the greatest number of exposures with IV line insertion being the most common activity during exposure. In a cross-sectional survey of 266 HCW in rural north India working in non-governmental health settings of 115 beds or less, nurses again had the highest reported number of exposures in the past year [12]. In our study, intern doctors (in their first year of training post-medical school) reported the highest incidence of first-time and repeat exposures. Several reasons may underlie interns high rates of exposure. Our study was in a teaching hospital where interns not nurses or other hospital personnel are expected to do routine blood draws, suturing and IV insertion procedures. Paired with their inexperience, long work hours and high volume of inpatient procedures puts interns at high risk. Other teaching hospitals have also reported the highest incidence of exposures among housestaff in both resource-contrained and resource-rich settings [19-22]. In South Africa, a survey of 98 interns found that 69% had one or more percutaneous exposures to blood, and similar to our study, these commonly occurred because of unexpected patient movement during procedures or during disposal of needles [20]. Data from a large study of US residents found that extended work duration and night work among interns is associated with increased risk of percutaneous injuries [19].

There are few studies regarding incidence of occupational exposures or PEP utilization in resource-contrained settings. A cross-sectional survey of Nigerian HCW at a teaching hospital found that 27% of HCWs had a needlestick in the past year with a rate of 0.6/PY [15]. The majority were dentists (100%) and surgeons (81%), followed by other physicians (31%), and nursing staff (31%). They reported patient movement, recapping, and accidental stick by colleague to be the major reasons for HCW exposure. A recent survey of HCW in Kenya found that there was low uptake of PEP (4% of needlestick injuries), and this was largely attributed to HCW fear of getting HIV tested as well as the perception that needlestick exposures were low risk for HIV [23]. In a small study in Malawi, PEP was reportedly underutilized with 19 of 29 HCW initiating PEP [24]. Many of these HCW were nurses and one of the reasons for low use of PEP was lack of awareness and fear of getting HIV tested.

Given that so many high-risk exposures occurred during or after a procedure, it is likely that improved use of PPE and introduction of safer medical devices (e.g. needleless systems and sharps with engineered sharps-injury protections) would reduce the occurrence of high risk exposure to contaminated sharps. A review of published studies has shown that these safer devices are associated with a reduction in percutaneous injury rates of 22–100% [25]. One of the major limitations to use of these devices is the cost. Efforts to reduce their cost by having local manufacturers produce them and having legislation to motivate demand for the use of these products is needed.

For the vast majority of cases, a stat PEP dose was administered within 24 hours suggesting quick assessment and good access to PEP was possible in our setting. Similar to other studies, we found rapid tests for HIV were more convenient and useful than ELISA-based testing for determining source status [26,27]. However, we did determine that PEP overuse was apparent for exposures judged to be low risk or the source was known to be HIV-negative. Only 66.1% of cases that received stat doses met CDC guidelines for which PEP is recommended. Furthermore, extended PEP regimens were used in 20 exposure cases even though the source tested HIV-negative. Designated clinicians indicated that one reason for the over-prescription of PEP stat doses and regimens was worker anxiety and misconceptions about the threat of HIV despite counseling by the on-call PEP physician. However, in the initial few weeks post-exposure many reviewed the indications for PEP and risk of HIV from available books and the internet and many subsequently realized that PEP was not indicated in them and hence discontinued. This was particularly true about the residents in Medicine, Obstetrics/Gynecology and Pathology. During the study, however, we observed that appropriate PEP utilization improved over time suggesting that there had been improved knowledge in the hospital setting regarding the true risk of bloodborne infection due to occupational exposure. Nevertheless, continued education about the true risk for HIV infection from exposures and guidelines for PEP use are needed to reduce HCW anxiety and optimize PEP utilization.

PEP coverage and compliance with an extended regimen in our study were similar to what has been reported in the US by the CDC [1]. Data from US National Surveillance System for Hospital Health Care Workers found that 6% of exposures were from HIV-infected sources and 63% of these HCW started PEP, with 54% taking it for 20 days or more[1]. In our study a higher proportion (15.7%) of exposures were from known HIV-infected sources. However, similar to US data, 65.9% of these cases began an extended PEP regimen, with 58.6% taking PEP for 20 days or more. Early discontinuation of extended PEP was often due toGI side effects of AZT. Ward attendants andsweepers more often discontinued PEP than interns or residents. While we do not have the exact reasons for this; we surmise it is likely because they did not perceive themselves to be at high risk for HIV infection and they were also less likely to truly have high risk exposures. They also may have been less knowledgeable about importance of adherence to PEP and may have experienced side effects of the PEP medications. It is important to note that indinavir was commonly used in the PEP combination. This drug was among the first PIs developed and used in early PEP regimens in the US but now is very rarely used as more tolerable and potent PIs are available and are recommended.

Since our incidence rates were based on reported incidents, it is likely that the rates are underestimated as several studies have found that many exposures are not reported. It is also possible that housestaff may have been more likely to be aware of the PEP program and more likely to report an exposure than other hospital staff but we would still expect that because housestaff are the most likely to perform procedures at a teaching hospital that they would have the highest exposure rates. Follow-up was inadequate and likely reflects the difficulty of relying on housestaff to return for follow-up visits on their own initiative. It is therefore possible there may have been some undetected HIV or Hep B seroconversions. A protocol for more active follow-up has been subsequently put into place. Another limitation was that our study took place at one large public teaching hospital and may not be generalizable to other hospital settings in India, where a formal program for reporting occupational exposures and providing PEP may be lacking.

## Conclusion

Our study suggests that a comprehensive program covering universal precautions, procedural training and sharps handling is imperative for HCW, in particular for interns at teaching hospitals. With their high exposure rates in mind, interns would likely benefit from greater supervision during procedures. Improved use of PPE, access to reliable rapid HIV testing, introduction of safer medical devices for procedures, and continued education regarding appropriate use of PEP are necessary to ensure optimal HCW safety in resource-limited settings such as India.

## Competing interests

The authors declare that they have no competing interests.

## Authors' contributions

AG designed the study, analyzed and interpreted the data and drafted the manuscript. SA assisted in the analysis, interpretation of data and drafting of the manuscript. JS assisted in the data collection, interpretation of the data and provided critical revisions for intellectual content. AK assisted in the acquisition and critical interpretation of the data. AB assisted in the acquisition and critical interpretation of the data. SB assisted in the acquisition and critical interpretation of the data. NG assisted in the analysis and interpretation of the data as well as drafting the statistical sections of the manuscript. RCB assisted in the conception and design of the design and provided critical revisions for intellectual content. ALK assisted in the study design, acquisition of data and provided critical revisions for intellectual content. All authors have given final approval of the version to be published.

## Pre-publication history

The pre-publication history for this paper can be accessed here:


